# Unveiling Oscillatory Behavior in the Electro-Oxidation of Ethanol on Nickel Electrodes

**DOI:** 10.1021/acs.jpcc.5c04664

**Published:** 2025-10-02

**Authors:** Paula B. Perroni, Germano Tremiliosi-Filho, Teko W. Napporn, Hamilton Varela

**Affiliations:** 1 São Carlos Institute of Chemistry, 42512 University of São Paulo PO Box 780, São Carlos, SP 13560-970, Brazil; 2 IC2MP UMR 7285 CNRS, 27077 Université de Poitiers, Poitiers 86073, Cedex 09, France

## Abstract

Ethanol derived from renewable sources is a carbon-neutral fuel, and its electro-oxidation offers a pathway for sustainable energy generation, reducing dependence on fossil fuels. Nickel, as a cost-effective alternative to noble metals, enhances the economic feasibility of large-scale applications in energy systems such as alkaline fuel cells and electrolyzers. This study presents an experimental investigation of ethanol electro-oxidation on nickel electrodes in alkaline media, focusing on electrocatalytic activity and the emergence of oscillatory kinetics. The results demonstrate that ethanol electro-oxidation is facilitated by NiOOH species, with the oxidation of ethanol identified as the rate-determining step. Cyclic voltammetry revealed that the conversion of Ni­(OH)_2_ to NiOOH plays a crucial role, and an increase in current density was observed, correlating with ethanol oxidation and the formation of additional anodic peaks. Stable potential oscillations persisted even under enhanced mass transport, indicating a dynamic interplay between the continuous formation and consumption of NiOOH species during ethanol oxidation at lower potentials and the oxygen evolution reaction at higher potentials.

## Graphical abstract

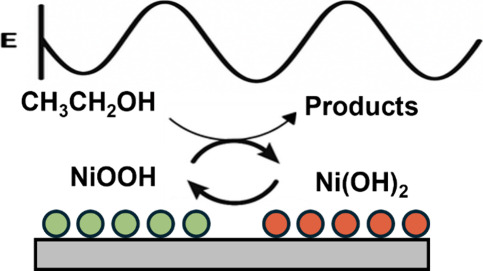

## Introduction

The electro-oxidation of organic molecules is of significant interest in the field of electrochemistry due to its wide range of applications. Among those molecules, ethanol stands out not only for its ability to reduce the energy required for water splitting in the so-called electrochemical reform but also as a sustainable biofuel derived from biomass.^1–3^ The electro-oxidation of ethanol can yield valuable products, making it an attractive candidate for both energy and chemical production applications.^4^ Overall, the electro-oxidation of small organic molecules involves the transfer of multiple electrons and the formation of adsorbed intermediates, which may block the electrode surface, slowing down the reaction rate and preventing complete oxidation to CO_2_. The selection of the catalyst plays a crucial role as it needs to stabilize these intermediates while also promoting efficient electron transfer, aiming at reducing the overpotential and improving the overall conversion.

Noble metals like Pt, Au, and Pd are often mentioned as effective catalysts for ethanol oxidation, both in acidic and alkaline environments.^5^ However, their limited availability and high cost restrict large-scale applications. The use of non-noble metals or transition metal oxides as catalysts has shown promise for various reactions in power generation, including Ni, Co, Cu, and Mn, among others.^6–8^ Many transition metals show potential for the ethanol electro-oxidation reaction (EEOR), with Ni-based electrodes gaining prominence due to their relatively low cost, corrosion resistance in alkaline media, and good catalytic response.^9^

Much research has been conducted on the oxidation of organics on Ni electrodes. In 1971, Fleischmann et al.^10,11^ conducted a study addressing the oxidation of organic molecules on Ni electrodes. In that work, they described that the process would not be governed by the adsorption of species on the electrode but rather by an interaction on the formed oxide film.^11^ In their analysis, the authors proposed that the conversion of nickel hydroxide to nickel oxyhydroxide would occur as a preliminary step, eq 1, resulting in the activation of the oxyhydroxide for oxidation, followed by organic oxidation, eq 2, as the sluggish path,

$$\mathrm{Ni}(\mathrm{OH}{)}_{2}+{\mathrm{OH}}^{-}⇌\mathrm{NiOOH}+H_{2}O+e^{-}$$ 1

$$\mathrm{NiOOH}+\mathrm{organic}\;\mathrm{molecule}\to \mathrm{Ni}{\left(\mathrm{OH}\right)}_{2}+\mathrm{product}$$ 2

The literature highlights the potential of nickel-based catalysts in the ethanol electro-oxidation reaction. Barbosa et al.^12^ discussed an autocatalytic mechanism, where β-NiOOH, formed at potentials above 1.3 V vs RHE, acts as the active species. They investigated the temperature dependency of the reaction and observed that it remains efficient across a wide temperature window, even at −15 °C, which underscores the robust activity of nickel under diverse thermal conditions. Recent work suggests that the presence of high-valence nickel species (Ni^3+^ and Ni^4+^), surface defects, and promoters facilitate key steps such as hydrogen atom and hydride transfer in the reaction mechanism.^13^ While some studies indicate that synergy between different metals can enhance the electrochemically active surface area and catalytic performance,^14^ the focus remains on understanding the detailed reaction mechanisms to optimize the activity and stability of nickel-based catalysts.

Intrinsically connected to the electrocatalysis of the oxidation of small organic molecules is the emergence of self-organization, mainly in the form of multistability and oscillatory reaction rates in the current and/or potential.^15–19^ Most reports are for the oxidation reaction on Pt surfaces, but there are also examples of oscillating behavior during the electro-oxidation of species such as urea and methanol on Ni. Vedharathinam and Botte^20^ reported the electro-oxidation of urea on a Ni electrode in alkaline medium and attributed the oscillations to the redox conversion Ni^2+^/Ni^3+^. Huang et al.^21^ concluded that the mechanism involved in the oscillatory electro-oxidation of methanol on Ni results from a combination of charge transfer, diffusion, and convective mass transfer.

In this article, we report on electrocatalytic oxidation of ethanol on Ni and in a KOH aqueous solution, focusing on the kinetic instabilities found under the galvanostatic regime. After an initial voltammetric characterization and discussion based on the current literature, we explore the observation of potential oscillations. Those are explained in terms of the Ni^2+^/Ni^3+^ redox transition and on the interplay between ethanol oxidation and oxygen evolution reaction (OER).

## Methodology

The working electrode used was a nickel plate (99.99%) with a geometric area of 1 cm^2^ exposed to the electrolyte. Before testing it, the electrode passed through a cleaning process by submerging it into an acid solution, containing 30% HNO_3_, in an ultrasonic bath for 30 min, followed by washing it in Milli-Q water and immersing it in acetone in an ultrasonic bath for another 30 min. After this procedure, the electrode was properly taken into the electrochemical cell previously purged with argon gas.

A three-compartment glass cell was used throughout the experiments. It was cleaned in sulfonitric acid (1:1 HNO_3_/H_2_SO_4_) followed by intensive washing with Milli-Q water and finally boiled in water for at least five times. All of the glasses used were stored in water.

A nickel mesh was used as the counter electrode, while a reversible hydrogen electrode (RHE) was used as the reference electrode, and all potentials discussed here are given with respect to this reference. All of the electrochemical measurements were performed using a potentiostatic/galvanostatic autolab PGSTAT302N from Metrohm, and the data were collected by Nova 2.1.7.

The methodology applied for area calculation was taken from Barbosa et al.^12^ and proposed by Machado and Avaca.^22^ It considers the charge associated with the reversible process in the anodic sweep in cyclic voltammetry. The electrochemical active surface area (*A*_ECSA_) was then calculated by eq 3:

$$Q=(q_{1\mathrm{MC}|\mathrm{Ni}(\mathrm{OH}{)}_{2}|}\times A_{\mathrm{ECSA}})+[A_{\mathrm{ECSA}}\times C_{\mathrm{dl}}\times (E_{f}-E_{i})]$$ 3

The current density from the formation of one monolayer of Ni­(OH)_2_ (*q*_1MC|Ni(OH)2|_) is equal to 514 μC cm^–2^,^22^ and the double layer capacitance (*C*_dl_) is equal to 20 μF cm^–2^,^23^ whereas *E*_f_ and *E*_i_ correspond to the final and initial potential of the considerate region, respectively.^22–24^ Cyclic voltammetries (CVs) were recorded between 0.85 and 1.55 V for Ni in electrolyte and 0.85 and 1.60 V in ethanol. Before the CVs, the potential was applied at 0.05 V for 1 min to reduce the previously formed oxide layer. After, oscillations were studied under galvanodynamic, at the rate of 10 mA s^–1^, and galvanostatic conditions.

All the measurements were performed in 1 mol L^–1^ of KOH (99.99% from Sigma-Aldrich), with no further purification step, and the study in ethanol was performed in 0.5 mol L^–1^ of the organic molecule (99.5% from JT. Baker) at 25 °C.

## Results and Discussion

### Voltammetric Responses

The system’s initial characterization is given in Figure 1a in terms of the consecutive cyclic voltammograms of the Ni electrode in a KOH aqueous solution at 50 mV s^–1^. As the cycles evolve, the charge of the oxidation peak increases, reflecting the progressive oxidation of metallic Ni and its conversion to the hydrated species NiO_*x*_H_*y*_, which is part of the reversible Ni­(OH)_2_/NiOOH reaction.^25^ The reversible peaks that appear in Figure 1a pertain to the transition of Ni^2+^ to Ni^3+^, as presented in eq 1.

**Figure 1:**
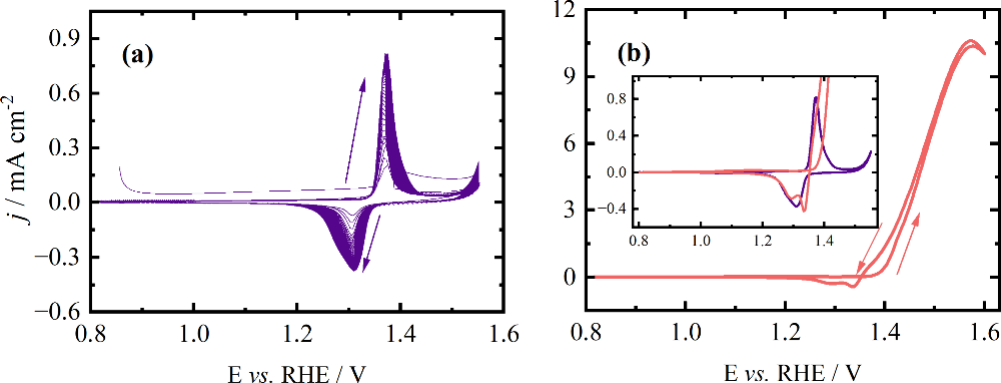
Cyclic voltammogram of the Ni electrode in (a) 1 mol L^–1^ KOH and (b) 1 mol L^–1^ KOH and 0.5 mol L^–1^ ethanol. Sweep rate: 50 mV s^–1^. The cycle in (b) corresponds to the 50th one.

During the cathodic sweep, two processes become evident in distinct potential regions. A well-defined peak at 1.34 V appears, followed by a period between 1.30 and 1.22 V. The appearance of the cathodic peak with the progression of scans combined with changes in the peaks’ position indicate changes in the crystallographic structures of the nickel oxide formed in the film.^26^ First, α-Ni­(OH)_2_ is formed and then is progressively converted to β-Ni­(OH)_2_ as the electrode is cycled, which is a more layer-organized phase.^27,28^ The cathodic process corresponds to the reduction of β-NiOOH to β-Ni­(OH)_2_.^29^

After studying the processes of Ni in KOH, the ethanol oxidation reaction was examined under identical conditions, and the results are given in Figure 1b. The increase in the anodic current indicates the oxidation of the organic compound, and this process prevails along the forward and most of the backward sweep.

The mechanism of ethanol oxidation on Ni electrodes begins with the oxidation of Ni­(OH)_2_ to NiOOH, which is widely reported as being the active species.^10,11,30^ The oxidation of ethanol is promoted by NiOOH species and is the rate-determining step of the reaction.^11,12^ A general mechanism of ethanol oxidation on Ni electrodes depends on NiOOH species, as proposed by Fleishmann et al.^11^

$$4\beta -\mathrm{NiOOH}+{\mathrm{CH}}_{3}{\mathrm{CH}}_{2}\mathrm{OH}+{\mathrm{OH}}^{-}\to 4\beta -\mathrm{Ni}{\left(\mathrm{OH}\right)}_{2}+{\mathrm{CH}}_{3}{\mathrm{COO}}^{-}$$ 4

The difficult cleavage of C–C bonds on this specific surface hinders the formation of CO_2_ as a final product, resulting instead in the formation of acetaldehyde and acetate.^12,31^ Unlike the well-known direct electron transfer by adsorption of the organic compound to the anode in metal catalysts,^32,33^ the process on Ni occurs via an alternative mechanism described as follows.^11^ Ethanol molecules adsorb on −OOH oxide sites, formed in eq 1, via the alcohol group, and then, the molecule undergoes initial dehydrogenation to acetaldehyde as the rate-determining step, eq 5. In eq 6, when the adsorbed intermediate is released, the −OOH species are reduced to −(OH)_2_, as described in eq 4. Acetaldehyde then reacts with OH^–^ via a nucleophilic addition resulting in an unstable intermediate that adsorbs onto another −OOH species, eq 7, which is oxidized to acetic acid, eq 8, and then is deprotonated and transformed into acetate, culminating in the production of the final product, c.f., eq 9.^11,12^ In the reaction equations, an asterisk (*) is used to specify the atom or group that is adsorbed on the electrode surface, distinguishing it from other atoms of the same element in the molecule.

$${\mathrm{CH}}_{3}{\mathrm{CH}}_{2}{\mathrm{OH}}_{\mathrm{sol}}+{\mathrm{OH}}^{-}\to {\mathrm{CH}}_{3}{\mathrm{CH}}_{2}O^{*}+H_{2}O+e^{-}$$ 5

$${\mathrm{CH}}_{3}{\mathrm{CH}}_{2}O^{*}+{\mathrm{OH}}^{-}\to {\mathrm{CH}}_{3}\mathrm{CH}O_{\mathrm{sol}}+H_{2}O+e^{-}$$ 6

$${\mathrm{CH}}_{3}\mathrm{CH}O_{\mathrm{sol}}+{\mathrm{OH}}^{-}\to {\mathrm{CH}}_{3}\mathrm{CH}O^{*}\mathrm{OH}$$ 7

$${\mathrm{CH}}_{3}\mathrm{CH}O^{*}\mathrm{OH}+{\mathrm{OH}}^{-}\to {\mathrm{CH}}_{3}{\mathrm{COOH}}_{\mathrm{sol}}+H_{2}O+e^{-}$$ 8

$${\mathrm{CH}}_{3}{\mathrm{COOH}}_{\mathrm{sol}}+{\mathrm{OH}}^{-}\to {\mathrm{CH}}_{3}{\mathrm{COO}}_{\mathrm{sol}}^{-}+H_{2}O$$ 9

The dependence of the current density on the scan rate was explored to understand if the limiting process of the reaction is mass transport or diffusion limited. Results are given in Figure 2, where voltammograms were recorded in the range between 5 and 1000 mV s^–1^ before and after the addition of ethanol. On Ni, both the anodic and cathodic peaks in the cyclic voltammograms rise as the scan rate increases, c.f., Figure 2a. Moreover, the anodic peak shifts toward more positive potentials, while the cathodic peak moves toward less positive ones. The separation of the peaks at 5 mV s^–1^ amounts to 52 mV (from 1.31 to 1.36 V), whereas at 1000 mV s^–1^, it amounts to 200 mV (from 1.26 to 1.46 V). This phenomenon may stem from an uncompensated ohmic drop enhanced by the increasing scan rate.^34^

**Figure 2:**
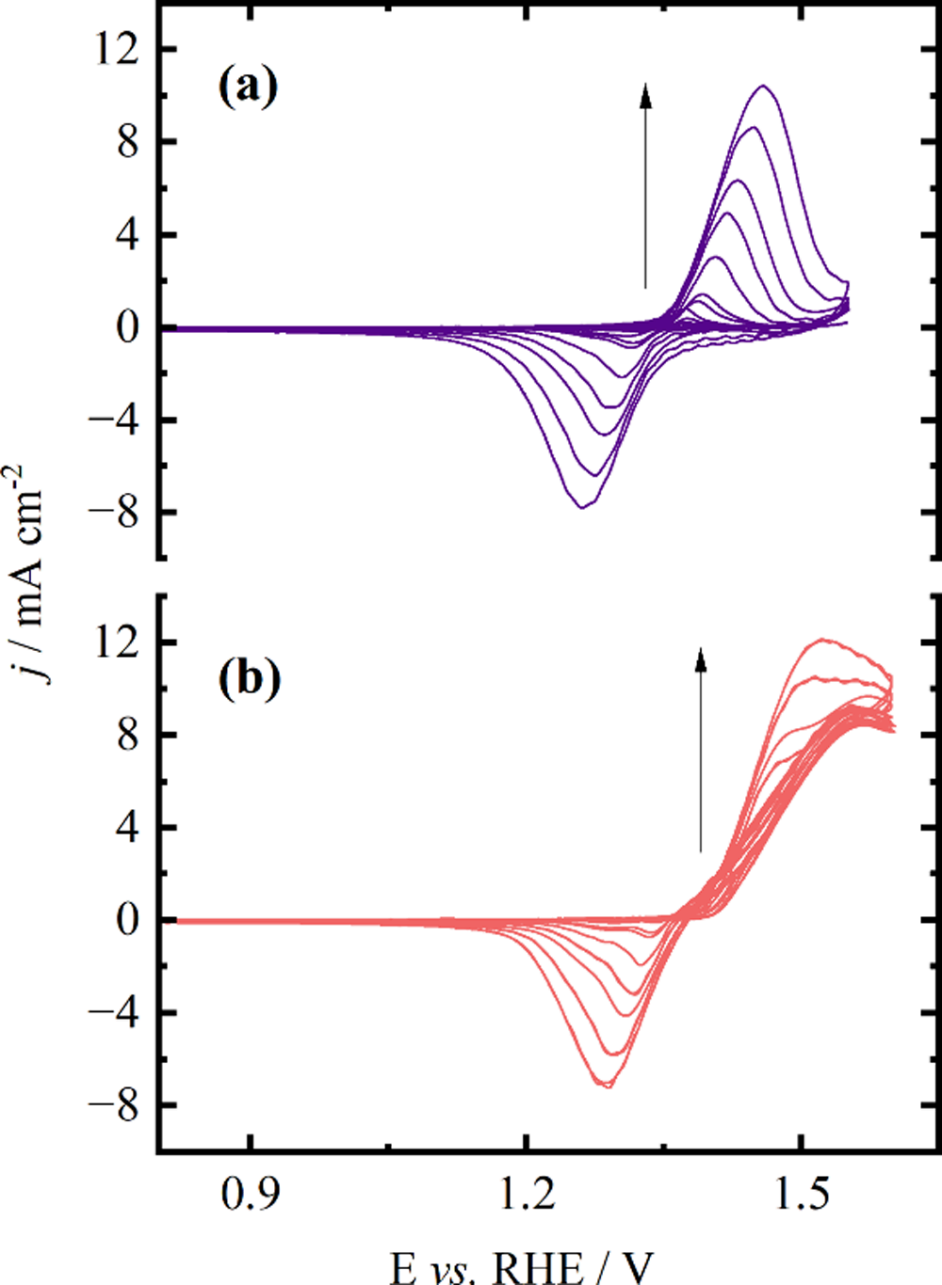
Cyclic voltammogram of Ni-only in 1 mol L^–1^ KOH (a) and in the presence of 0.5 mol L^–1^ of ethanol (b) at various potential sweep rates of 5, 10, 20, 50, 75, 100, 200, 350, 500, 750, and 1000 mV s^–1^.

At scan rates up to 100 mV s^–1^, the current peak shows linear dependence with the scan rate, demonstrated in Figure S1a, indicating charge transfer limitation and normal behavior for a reversible reaction. As the scan rate increases, the current peak increases linearly with the square root of the scan rate, indicating a diffusion-controlled process, c.f., Figure S1b.^35,36^ At higher scan rates, however, capacitive contributions and ohmic drop may influence the response, although the trend remains charge transfer-dominated.

When ethanol is present, the reduction peak is nearly undetectable at lower scan rates but becomes more pronounced as the scan rate increases, as shown in Figure 2b. This observation suggests that the conversion of Ni^2+^ to Ni^3+^ is faster than ethanol oxidation, in agreement with previously published data.^10,11^ At low scan rates, the absence of the reduction peak can be explained by the interaction between EOR and the formation of Ni^3^^+^. A plausible hypothesis is that, at low scan rates, the available time allows ethanol oxidation to occur more completely, consuming the formed Ni^3^^+^ species. This means that when the reverse scan occurs, there is less Ni^3^^+^ available to be reduced back to Ni^2^^+^, resulting in the absence of the reduction peak.^26,34^

As the scan rate increases, the formation of Ni^3^^+^ becomes faster than the oxidation of ethanol, and less Ni^3^^+^ is consumed during the latter process. This results in a greater amount of Ni^3^^+^ present during the reverse scan, leading to the emergence and increase of the reduction peak. Therefore, the absence of the reduction peak at low scan rates may be because ethanol oxidation efficiently consumes the formed Ni^3^^+^ species before they can be reduced back to Ni^2^^+^ in the reverse scan.

Between 5 and 20 mV s^–1^, the current peak diminishes as the scan rate rises, and no linearity is observed with the scan rate. Beyond 20 mV s^–1^, however, the peak current begins to increase, exhibiting a more linear relationship with the scan rate rather than following the square root of the scan rate, as can be seen by comparing the *R* ^2^ from the linear regression (0.97 and 0.93, respectively) in Figure S1c,d. These observations suggest that the reaction is a limited charge transfer in higher scan rates.^34,37^

The unexpected behavior at low scan rates can be attributed to the charge transfer limitation in the formation of Ni^3^^+^, as discussed for the linearity of the current peak and scan rate in Ni-only in KOH. Since the reaction is controlled by the charge transfer, the formation of Ni^3^^+^ cannot keep up with the scan rate, potentially leading to the accumulation of intermediates formed during the reaction and the unavailability of active species to complete the reaction.

As the scan rate increases, a notable new oxidation peak emerges on the positive sweep, as illustrated in Figure S2. The height of this peak is linearly dependent on the scan rate, as demonstrated in Figure 4b. This phenomenon may be linked to the formation of NiOOH, as the rapid scan prevents ethanol from reacting properly. Consequently, the peak becomes more pronounced and increases with the rising scan rate.

### Potential Oscillations

Along a quasi-stationary galvanodynamic sweep (Figure S2), oscillations were recorded during ethanol oxidation on a Ni electrode. Potential oscillations were observed from 2.4 to 2.7 mA cm^–^^2^, with lower and upper potential limits between 1.42 and 1.49 V. From the galvanodynamic curve (Figure 4b), a current density of 2.52 mA cm^–2^ was chosen and applied (Figure 3). This value corresponds to the average current density at which oscillations appear. The system oscillates after an induction period of nearly 2 h, and this was attributed to surface conditioning prior to oscillation onset. During this period, NiOOH progressively forms and stabilizes, and a steady-state coverage of adsorbed intermediates builds up, both of which are necessary to trigger the oscillations. The potential began to oscillate and persisted for more than 1 h, after which it stabilized at ∼1.55 V. Between cycles, a constant potential period was followed by a potential jump, characterized by a slow decrease and then a rapid decrease to lower potential values. As time progressed, the duration of the high-potential phases increased, followed by shorter cycles. The amplitude of the oscillations remained at about 10 mV, with an average frequency of 97 mHz. Small modulations appeared in the cycles just before the potential increase and decreased over time.

**Figure 3:**
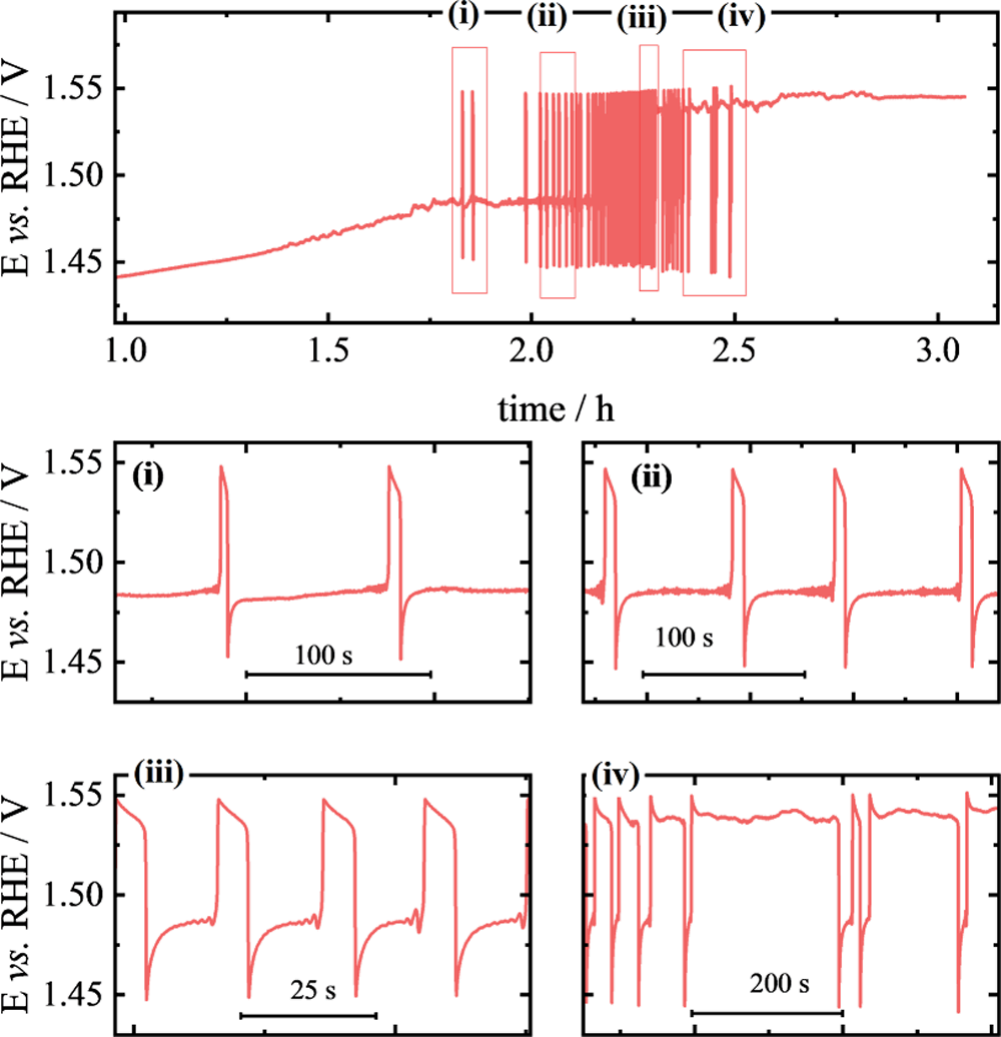
Potential oscillations during chronopotentiometry obtained in 0.5 mol L^–1^ of ethanol on 1 mol L^–1^ of KOH at applied current density of 2.52 mA cm^–2^. Parts (i)–(iv) correspond to regions given in the upper curve.

Potential oscillations have been previously reported for the electro-oxidation of urea and methanol on polycrystalline Ni.^20,21^ In both cases, the oscillations were attributed to Ni­(OH)_2_/NiOOH transitions, although the underlying mechanisms differ. For methanol,^21^ the authors suggest the involvement of oxygen in the oscillations since the potential reaches the OER region. The proposed mechanism involves methanol scarcity at the metal surface, as the applied current exceeds the limiting stationary oxidation current. This scarcity causes the potential to increase to the OER region, where oxygen bubbles promote the local stirring, thus enhancing the mass transport near the surface, which in turn helps replenish methanol at the surface. During this process, NiOOH is reduced to Ni­(OH)_2_ due to the methanol oxidation and then reoxidized to NiOOH at high potential.

In contrast, a different mechanism was proposed by Vedharathinam and Botte^20^ for the electro-oxidation of urea. In particular, the potential does not reach the OER region, and oscillations persist even under stirring, indicating that a mass transfer limitation does not play a critical role. Instead, oscillations result from the Ni­(OH)_2_/NiOOH conversion, with the potential increase associated with Ni^2^^+^/Ni^3^^+^ reactions and the decrease corresponding to the reverse reaction.

**Figure 4:**
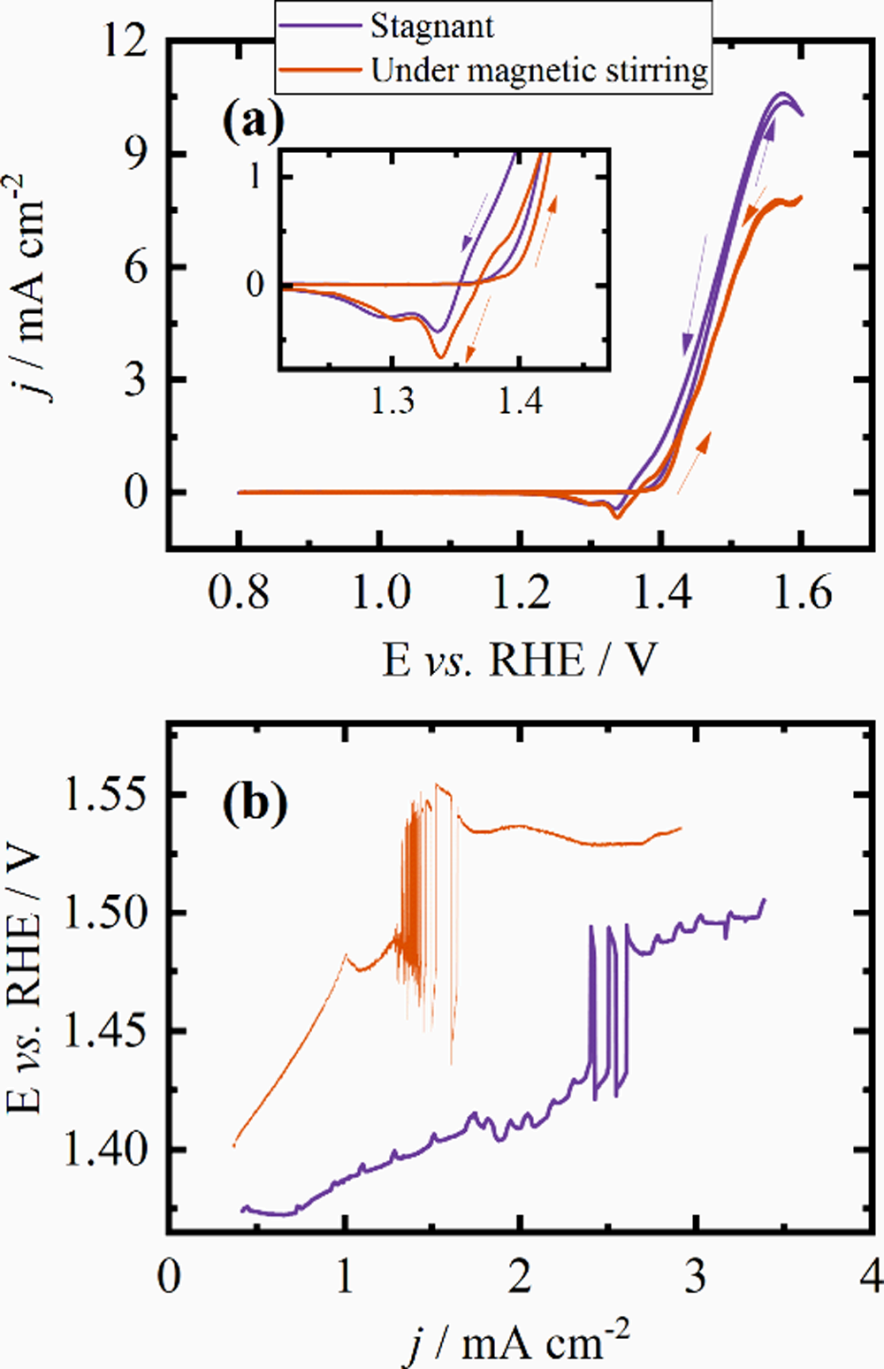
(a) Cyclic voltammogram of 0.5 mol L^–1^ in 1 mol L^–1^ of KOH in 50 mV s^–1^ for a stagnant solution and subject to magnetic stirring of 1000 rpm. Inset: cathodic peak. (b) Galvanodynamic at 10 μA s^–1^.

Figure 4a shows the cyclic voltammograms of ethanol oxidation under different stirring conditions. Despite the enhanced diffusion of species from the solution to the electrode surface due to stirring, a decrease in the oxidation current density is observed—from 10.4 to 7.8 mA cm^–^^2^—along with an increase in the cathodic peak.

The oxidation of ethanol in the oxide surface is sluggish, and stirring can remove reactive intermediates from the surface, reducing the reaction efficiency. The higher cathodic peak density current corroborates this hypothesis. As less ethanol is oxidized, less Ni^3+^ species are reduced during the reaction and there are more of these species to be reduced to Ni^2+^ in the cathodic process.^34^ This difficulty in ethanol oxidation may explain the oscillations occurring at higher potentials.

We also investigated the potential oscillations during a galvanodynamic experiment conducted under magnetic stirring of the solution (at 1000 rpm) to assess the influence of mass transport. As shown in Figure 4b, oscillations occur at lower current densities under stirring (1.30 and 1.64 mA cm^–2^) compared to the stationary mode (2.4–2.6 mA cm^–2^). The potential amplitude under stirring is shifted to higher values, from 1.47 to 1.52 V in the beginning of the oscillatory region, which evolves to 1.43–1.55 V, whereas under stationary conditions, it remains between 1.42 and 1.50 V. This upward shift in the oscillatory potential window under stirring can be explained by a mechanistic change in the Ni­(OH)_2_/NiOOH cycle. Stirring accelerates the chemical reduction of NiOOH by ethanol, increasing the time spent in the high-potential region, where Ni­(OH)_2_ is electrochemically regenerated, thus shifting the oscillations to higher average potentials despite their earlier onset in the current density.

These results suggest that while mass transport affects the onset and evolution of oscillations—lowering the current density and slightly increasing the potential range, it does not suppress the oscillations. Thus, the oscillatory dynamics are influenced, but not critically determined, by transport of ethanol to the electrode surface.

Scheme 1 displays the typical oscillatory profile and details of the mechanism proposed, according to the potential time-trace:

**Scheme 1:**
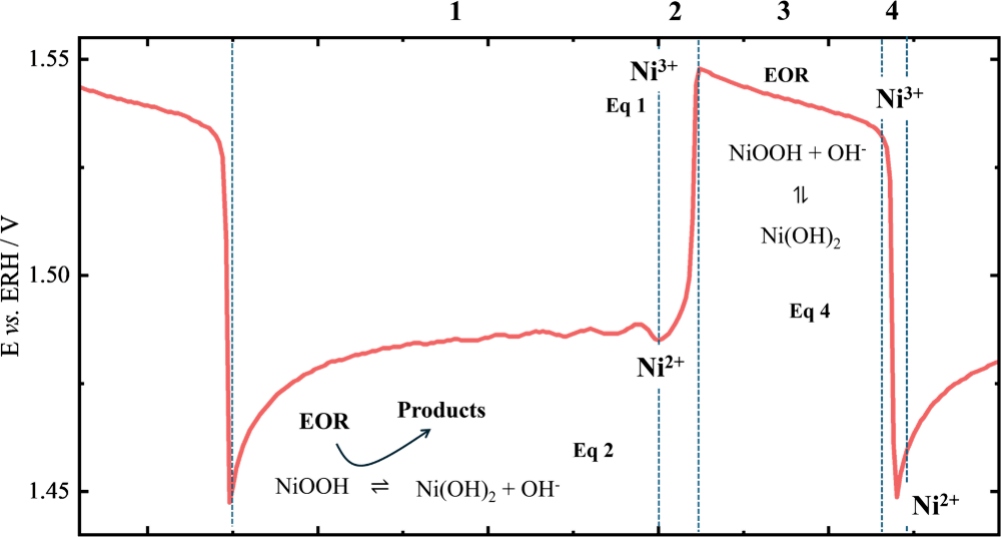
Proposed Mechanism That Originates the Oscillations During 0.5 mol L^–1^ of Ethanol Oxidation in 1 mol L^–1^ of KOH in the Nickel Electrode

From 1.45 to 1.48 V: The potential slowly increases, and small modulations appear due to interactions of the reaction of the intermediates from the oxidation of ethanol with the electrode surface. Active NiOOH species form and react slowly with ethanol, as in eq 4, converting to Ni­(OH)_2_, which is inactive.
From 1.48 to 1.55 V: The potential increases rapidly as NiOOH is electrochemically regenerated from Ni­(OH)_2_, via eq 1.
From 1.55 to 1.53 V: The potential slowly decreases as NiOOH is converted to Ni­(OH)_2_ while OER takes place, via eq 4.^11^
From 1.53 to 1.45 V: There is an abrupt potential decrease as NiOOH is converted to Ni­(OH)_2_, i.e., the reverse of eq 1.^11^

The cycle repeats because, even in step 4, the potential remains high enough for Ni­(OH)_2_ to convert back to NiOOH, allowing new ethanol species to interact with the active NiOOH and continue the reaction.

The proposed mechanism suggested that the potential oscillations from ethanol oxidation on the Ni electrode originate from the consumption of −OOH species from ethanol oxidation, which leads to NiOOH conversion to Ni­(OH)_2_.

The oscillatory behavior observed during ethanol electro-oxidation on Ni shows similarities with the cases reported for methanol and urea and also presents important differences. In all three systems, the Ni­(OH)_2_/NiOOH redox transition plays a central role in the oscillations. However, in the case of urea, Vedharathinam and Botte^20^ proposed that the oscillations result exclusively from this redox conversion, with no involvement of oxygen evolution or influence of mass transport. For methanol, Huang et al.^21^ suggested a mechanism, where the oscillations are triggered when the applied current exceeds the stationary oxidation current, leading to methanol depletion at the surface. The potential then increases to the OER region, where the formation of oxygen bubbles improves mass transport, restoring methanol to the surface and allowing the cycle to continue.

In our case, ethanol oxidation also leads to potential oscillations in a potential range similar to that of methanol oxidation, but the characteristics are different. The oscillations are not limited by mass transport, but the potential onset is higher under stirring than under stationary conditions. Additionally, the system requires a longer induction period under stationary conditions. These features point to a sluggish oxidation of ethanol on the Ni oxide surface, as supported by the CVs under stirring conditions, where the oxidation current decreases and the cathodic peak increases. This suggests that ethanol reacts less efficiently with NiOOH species, leading to the accumulation of oxidized species on the surface. Therefore, the mechanism we propose shares elements with both literature cases and highlights specificities of ethanol that cannot be neglected. This indicates that the identity of the molecule plays an important role in defining oscillatory behavior, and the mechanism is not fully general.

Finally, it is important to note that the proposed mechanism likely involves not only surface redox transitions but also bulk processes, possibly including the intercalation of cations into the Ni-based structure. These aspects will be explored in detail in a forthcoming study.

## Conclusions

- Ethanol electro-oxidation on nickel electrodes in alkaline media exhibits stable potential oscillations, even under magnetic stirring, indicating that mass transport is not the sole limiting factor.
- The oscillations arise from the dynamic interplay between Ni­(OH)_2_/NiOOH redox transitions and ethanol oxidation, suggesting a cyclical modulation of surface reactivity due to the dynamic conversion between NiOOH and Ni­(OH)_2_.
- This study contributes to the fundamental understanding of kinetic instabilities in electrocatalysis, particularly for non-noble metal systems like nickel.
- Future work should explore the contribution of bulk processes, such as cation intercalation into the Ni-based oxide layer, as well as the development of mathematical models that capture the interplay between ethanol oxidation and surface redox dynamics.

## Supplementary Material



## Notes

Figure S1 presents the dependence of peak current density on scan rate for the ethanol oxidation reaction and Figure S2 shows the analysis of peak current versus scan rate, supporting the discussion on reaction kinetics (PDF)
