# Supplementary material for: Unveiling Oscillatory Behavior in the Electro-Oxidation of Ethanol on Nickel Electrodes
Source: J Phys Chem C Nanomater Interfaces. 2025 Oct 2;129(41):18496–502. doi: 10.1021/acs.jpcc.5c04664 (PMC12536493; doi:10.1021/acs.jpcc.5c04664)
Supplement: Supplementary file 1 [file jp5c04664_si_001.pdf]

Supporting Information

for

Unveiling Oscillatory Behavior in the Electro-oxidation of Ethanol on Nickel Electrodes

Paula B. Perroni,[1] Germano Tremiliosi-Filho,[1] Teko W. Napporn,[2] Hamilton Varela,[1,]*

[1] São Carlos Institute of Chemistry, University of São Paulo, PO Box 780, 13560-970, São Carlos, SP, Brazil

[2] Université de Poitiers, IC2MP UMR 7285 CNRS, 86073 Poitiers Cedex 09, France

*corresponding author (HV): hamiltonvarela@usp.br

Table of contents

Figure S1: Dependence of density current peak with scan rate.

Figure S2: Peak current analysis vs. scan rate in of ethanol oxidation.

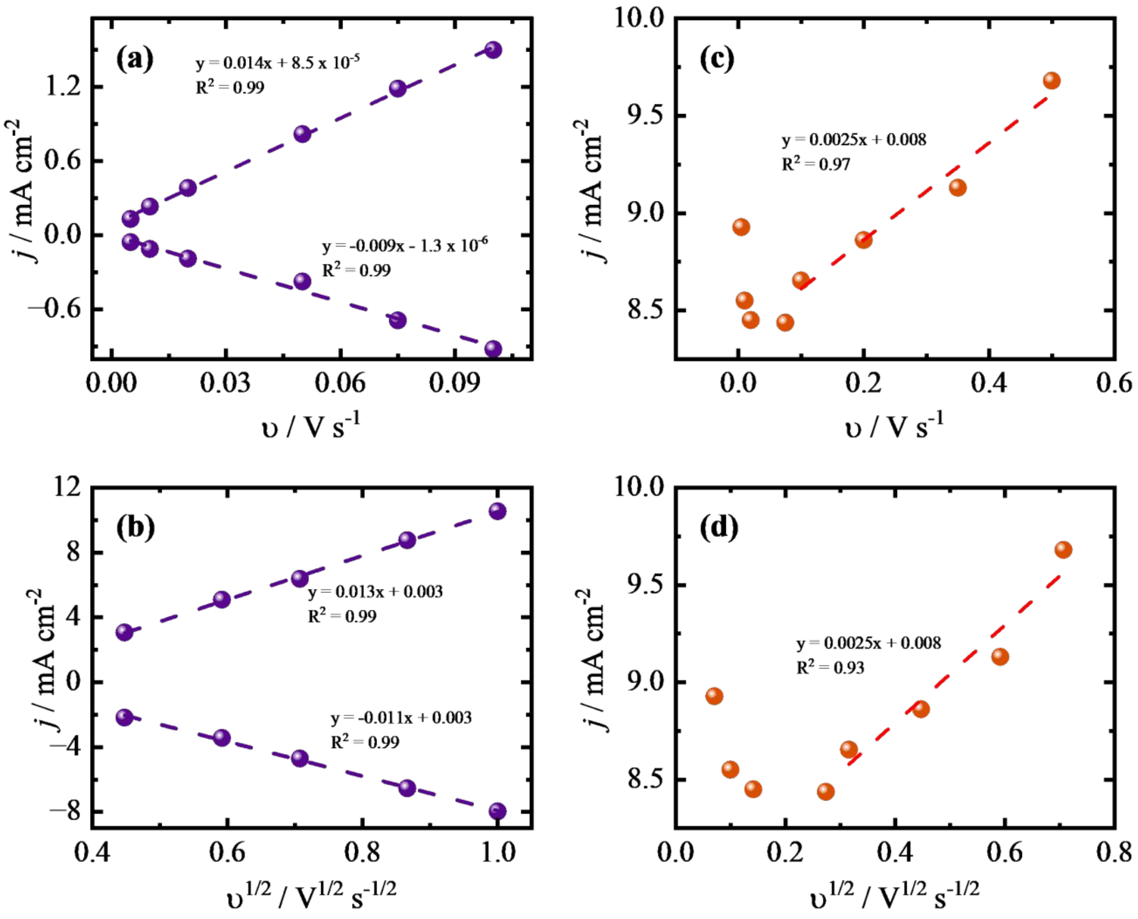

Figure S1: Dependence of density current peak with scan rate of anodic and cathodic peaks from the voltammograms in Fig. 2. Panels (a) and (b) show the linear regression of the current peaks for Ni in 1 mol L^{-1} of KOH as a function of the scan rate and the square root of scan rate, respectively. (d) and (e) show the linear regression of the peak currents of 0.5 1 mol L^{-1} ethanol, also as a function of the scan rate and the square root of the scan rate, respectively.

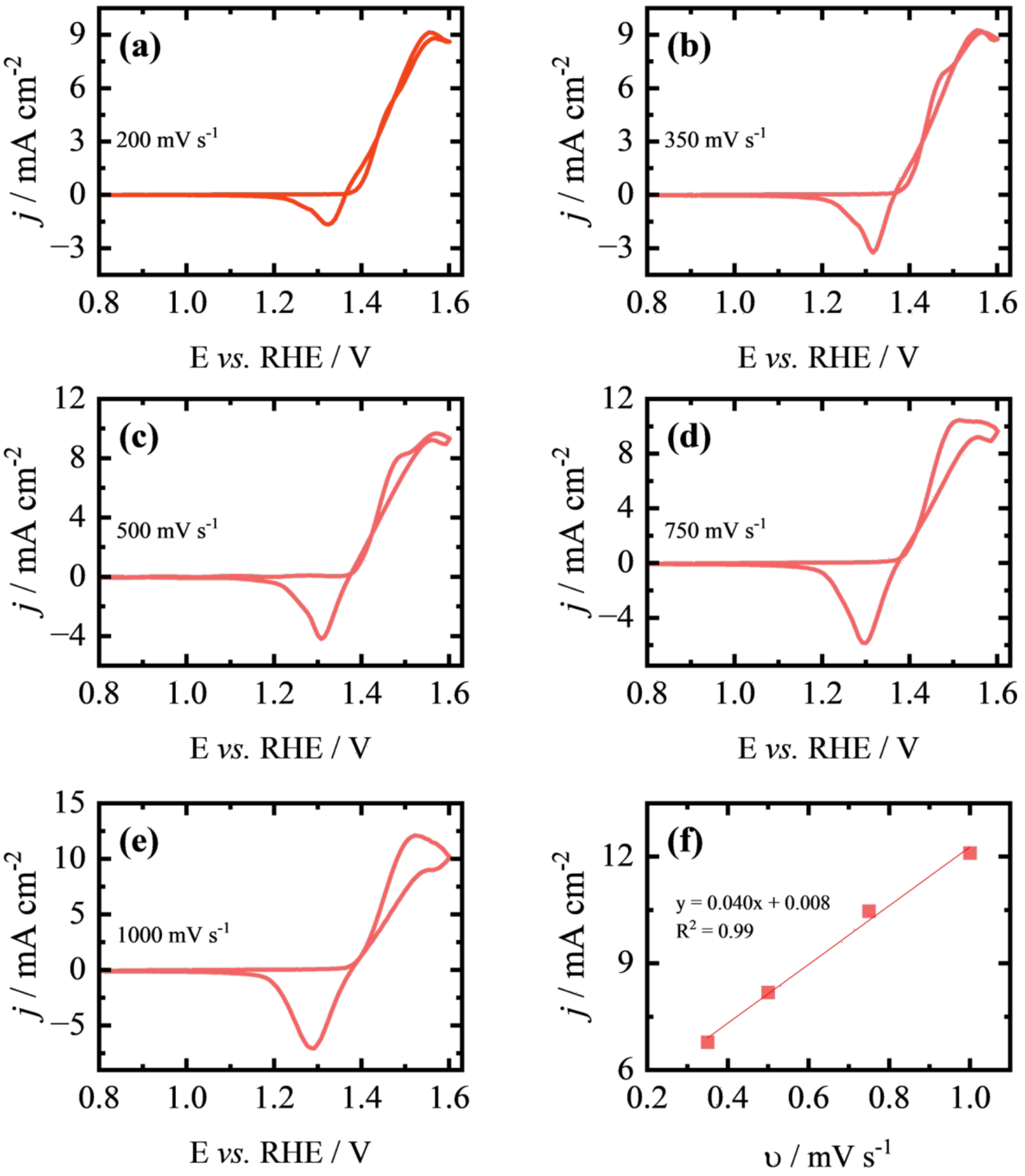

Figure S2: Cyclic voltammetry of 0.5 mol L^{-1} in 1 mol L^{-1} of KOH in a Ni electrode at various scan rates: (a) 200 mV s^{-1}; (b) 300 mV s^{-1}, (c) 500 mV s^{-1}, (d) 750 mV s^{-1}, (e) 1000 mV s^{-1}. (f) represents the current density peak of the new oxidation peak dependence with the scan rate.
